# Simultaneous Determination of Aesculin, Aesculetin, Fraxetin, Fraxin and Polydatin in Beagle Dog Plasma by UPLC-ESI-MS/MS and Its Application in a Pharmacokinetic Study after Oral Administration Extracts of *Ledum palustre* L.

**DOI:** 10.3390/molecules23092285

**Published:** 2018-09-07

**Authors:** Zhibin Wang, Wenbo Zhu, Hua Liu, Gaosong Wu, Mengmeng Song, Bingyou Yang, Deqiang Yang, Qiuhong Wang, Haixue Kuang

**Affiliations:** 1 Key Laboratory of Chinese Materia Medica (Ministry of Education), Heilongjiang University of Chinese Medicine, 24 Heping Road, Xiangfang District, Harbin 150040, China; wzbmailbox@126.com (Z.W.); zwb931014@126.com (W.Z.); 15776992843@163.com (H.L.); gaosong0910@yahoo.com (G.W.); a1467219165@163.com (M.S.); ybywater@163.com (B.Y.); eltyhappy@163.com (D.Y.); qhwang668@sina.com (Q.W.); 2 School of Traditional Chinese Medicine, Guangdong Pharmaceutical University, 232 Outer Ring Road, University Town, Guangzhou 510006, China

**Keywords:** *Ledum palustre* L., UPLC-ESI-MS/MS, pharmacokinetics

## Abstract

A rapid, simple and sensitive ultra-performance liquid chromatography-electrospray-ionization-tandem mass spectrometry (UPLC-ESI-MS/MS) method was developed and validated for the simultaneous determination of aesculin, aesculetin, fraxetin, fraxin and polydatin in beagle dog plasma for the first time. Plasma samples were pretreated by protein precipitation with methanol. Chromatographic separation was performed on an Acquity UPLC HSS T3 C18 column (2.1 mm × 100 mm, 1.8 μm) with gradient elution at a flow rate of 0.4 mL/min, using a mobile phase consisting of 0.1% formic acid (A) and acetonitrile (B). The analytes and IS were detected by multiple reaction monitoring (MRM) via negative ion mode with ion transitions of *m*/*z* 339.1–*m*/*z* 176.8 for aesculin, *m*/*z* 176.8–*m*/*z* 88.9 for aesculetin, *m*/*z* 206.8–*m*/*z* 192.1 for fraxetin, *m*/*z* 369.1–*m*/*z* 206.9 for fraxin, *m*/*z* 389.1–*m*/*z* 227.0 for polydatin and *m*/*z* 415.2–*m*/*z* 295.1 for puerarin. This method was validated according to the FDA guidelines and the results met the requirements of analysis. The calibration curves of analytes were linear with correlation coefficients more than 0.9980. The intra- and inter-day precisions were less than 15% and the accuracy was within ±15%. The maximum plasma concentration (Cmax) of aesculin, aesculetin, fraxetin, fraxin and polydatin was 46.75 ± 7.46, 209.9 ± 57.65, 369.7 ± 48.87, 67.04 ± 12.09 and 47.14 ± 12.04 ng/mL, respectively. The time to reach the maximum plasma concentration (Tmax) was 1.32 ± 0.38 h for aesculin, 1.03 ± 0.27 h for aesculetin, 0.94 ± 0.23 h for fraxetin, 0.83 ± 0.18 h for fraxin and 1.15 ± 0.15 h for polydatin. The results indicated that the absorption of aesculin might be slow in beagle dog plasma. This method was successfully applied for pharmacokinetics in beagle dog plasma after oral administration of the extracts of *Ledum palustre* L. at a dosage of 0.27 g/kg.

## 1. Introduction

*Ledum palustre* L. is an evergreen low shrub, which belongs to the Ericaceae family and is widely distributed in northern Europe, central Europe, northern Asia, America and the northeast of China. The ointment and decoction of *L. palustre* have been used to treat menxenia, infertility and gastric ulcers in folk for a long time. Not only that, it also plays an important role in treating insect bites, arthrosis, and rheumatism [1,2,3]. Previous studies have reported that *L. palustre* contains various types of components, such as coumarins, flavonoids, triterpenes, volatile oils and hydroxycinnamic acids [4]. The aesculin, aesculetin, fraxetin and fraxin belong to coumarins, and polydatin is an active stilbene compound. The five components isolated from *L. palustre* are the main active ingredients in natural herbs [5,6]. Pharmacological studies have revealed that the five components have several similar pharmacological activities, including anti-inflammatory, anti-oxidant, anti-tumor, anti-viral, and so on [7,8,9,10,11,12]. In particular, polydatin possesses extensive cardiovascular pharmacological activity, such as cardio-myocyte protection, vascular smooth muscle dilation, anti-thrombosis, etc. [13,14].

To investigate the pharmacokinetic characteristics of aesculin, aesculetin, fraxetin, fraxin and polydatin, several analytical methods based on chromatographic techniques for single or simultaneous quantification of analytes have been reported in recent years. High-performance liquid chromatography with ultraviolet (HPLC-UV), high-performance liquid chromatography quadrupole time of flight tandem mass (HPLC-QTQF-MS/MS), high-performance liquid chromatography with fluorescence detection (HPLC-FD), liquid chromatography tandem mass spectrometry (LC-MS/MS) and high-performance liquid chromatography tandem mass spectrometry (HPLC-MS/MS) methods were implemented for the determination and quantitation of aesculin, aesculetin, fraxetin, fraxin and polydatin in rat plasma and rabbit plasma [15,16,17,18,19,20,21]. In addition, only a single report was retrieved online which described the pharmacokinetics of polydatin in beagle dog plasma [22]. The gastric emptying time and pH value of gastric fluid in beagle dogs was similar to humans under a fasting state. The detection results of drug in beagle dog can be used to analyze change regulation in the human body. Therefore, it is important to develop a method for simultaneous determination of the five components in beagle dog plasma.

A rapid, sensitive and simple ultra-performance liquid chromatography-electrospray-ionization-tandem mass spectrometry (UPLC-ESI-MS/MS) method for the simultaneous determination of aesculin, aesculetin, fraxetin, fraxin and polydatin was developed and validated in this paper. This method was successfully applied to pharmacokinetics of the five components above after oral administration of extracts of *L. palustre* in beagle dog for the first time. The results of this study will provide useful information for further experimental research and clinical application. 

## 2. Results and Discussion

### 2.1. Optimization of UPLC-ESI-MS/MS Conditions 

The mobile phase was a decisive factor in the chromatographic analysis, which could affect the separation and ionization. Different solvent combinations, including water-acetonitrile and water-methanol with formic acid, were required to be tested for perfect peak shape, high response and stable retention time. It was found that the water-acetonitrile system possessed good separation efficiency with lower noise, and the retention time of the analytes was more stable than the water-methanol system. Higher signal response and sharp peaks of analytes were observed after addition of 0.1% formic acid into the mobile phase, and the sensitivity of detecting was improved. Therefore, 0.1% formic acid-acetonitrile was employed as the final mobile phase with gradient elution. The total test time was 5 min.

**Figure 1:**
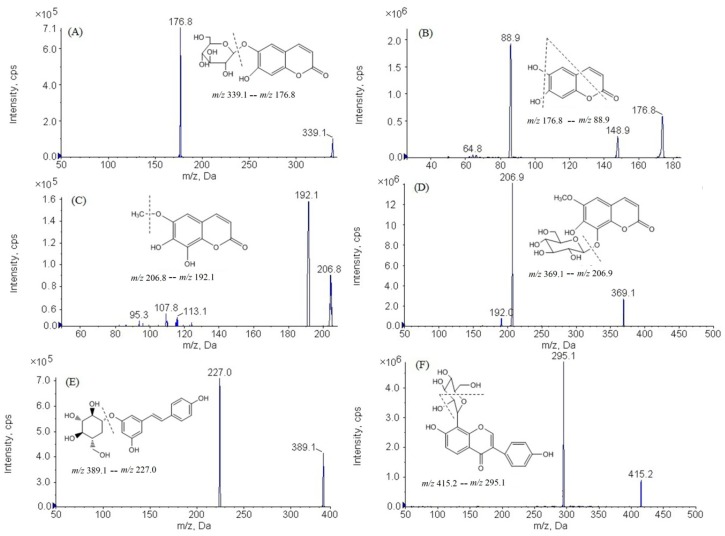
Chemical structures and product ion mass spectra of analytes and IS. (**A**): aesculin; (**B**): aesculetin; (**C**)**:** fraxetin; (**D**): fraxin; (**E**)**:** polydatin; (**F**): puerarin.

The analytes and IS were determined in both negative and positive ion mode, the signal sensitivity and reproducibility were better in negative ionization mode than in positive mode. Therefore, the negative ion mode was selected for the quantification. On the basis of the full-scan spectra shown in Figure 1, the deprotonated molecular ions [M − H]^−^ were selected as parent ions because of their high response. The declustering potential (DP) and collision energy (CE) of analytes and IS were optimized in order to obtain product ion. In the process of optimization, the most abundant and stable fragment was *m*/*z* 176.8 for aesculin, *m*/*z* 88.9 for aesculetin, *m*/*z* 192.1 for fraxetin, *m*/*z* 206.9 for fraxin, *m*/*z* 227.0 for polydatin, and *m*/*z* 295.1 for puerarin. Therefore, the ion transitions of the analytes were *m*/*z* 339.1–*m*/*z* 176.8 for aesculin, *m*/*z* 176.8–*m*/*z* 88.9 for aesculetin, *m*/*z* 206.8–*m*/*z* 192.1 for fraxetin, *m*/*z* 369.1–*m*/*z* 206.9 for fraxin, *m*/*z* 389.1–*m*/*z* 227.0 for polydatin and *m*/*z* 415.2–*m*/*z* 295.1 for puerarin. The optimized parameters and MRM transitions of the analytes and IS can be found in Table 1. 

### 2.2. Extraction Method 

The extraction reagents play an important role in extraction recovery and matrix effect. To select the appropriate extraction method, the protein precipitation (methanol and acetonitrile) and liquid-liquid extraction (LLE) (dichloromethane, ethyl acetate, acetone, mixture of ethyl acetate-acetone at different ratios) methods were evaluated by treatment of biological samples. The liquid-liquid extraction method was abandoned due to its low extraction recovery and high matrix interferences. Protein precipitation with acetonitrile has a high extraction recovery; however, strong interferences were observed. The recovery of analytes and IS were higher and more stable with methanol, with no obvious interferences. Therefore, methanol was chosen as the extraction solvent.

### 2.3. Method Validation

#### 2.3.1. Selectivity and Specificity 

**Figure 2:**
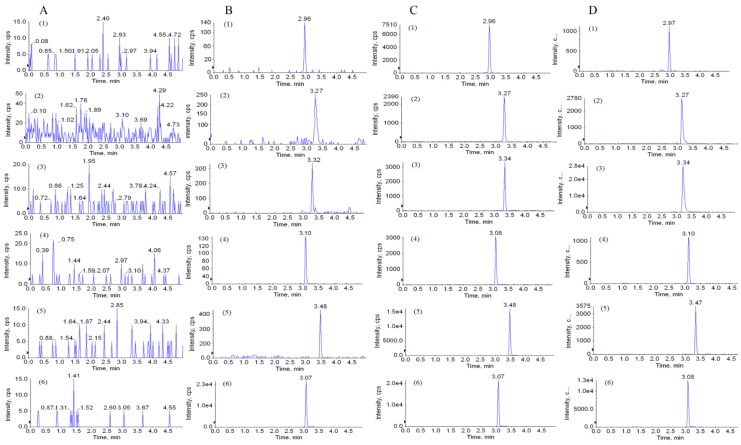
Typical MRM chromatograms of aesculin (1), aesculetin (2), fraxetin (3), fraxin (4), polydatsin (5), puerarin (6). Blank plasma from six beagle dogs (**A**); spiked plasma samples with the analytes and IS at LLOQ level (**B**); spiked plasma samples with the analytes and IS at QCM (**C**); plasma samples from beagle dog 1 h after oral administration of the extract of *L. palustre* (**D**).

The six beagle dogs’ blank plasma, spiked plasma samples with the analytes and IS at lowest limit of quantification (LLOQ) level, spiked plasma samples with the analytes and IS at quality control middle (QCM) level, and plasma samples 1 h after oral administration of the extract of *L. palustre* are displayed in Figure 2. The retention time of aesculin, aesculetin, fraxetin, fraxin, polydatin and IS were 2.96, 3.27, 3.34, 3.10, 3.48 and 3.07 min, respectively. No apparent interferences were observed from the endogenous substance during the retention time of the analytes and IS. 

#### 2.3.2. Linearity and Sensitivity 

The linearity status of analytes such as typical regression equations, correlation coefficients and linear ranges are displayed in Table 2. The results show that calibration curves for the five analytes were linear in the range of 0.50–97.50 ng/mL for aesculin, 1.09–209.2 ng/mL for aesculetin, 2.15–412.8 ng/mL for fraxetin, 1.25–241.0 ng/mL for fraxin, and 0.50–97.50 ng/mL for polydatin in beagle dog plasma, with correlation coefficients greater than 0.9980. The LLOQs of aesculin, aesculetin, fraxetin, fraxin and polydatin were 0.50, 1.09, 2.15, 1.25 and 0.50 ng/mL, respectively. 

#### 2.3.3. Precision and Accuracy 

The measurement results for precision and accuracy on LLOQ and QC samples (low, middle and high) are shown in Table 3. The intra-day and inter-day precisions of the analytes were lower than 16.1%, and the accuracy ranged from −5.2% to 16.5%. All values were within acceptable limits, indicating that the method described above has better repeatability and reproducibility.

#### 2.3.4. Extraction Recovery and Matrix Effect 

The extraction recovery of the five analytes at low, middle and high concentrations ranged from 80.10% to 98.54%, with RSD < 10.2%. The results indicate that the extract method of protein precipitation is suitable for the determination. The matrix effects of the analytes at the three concentrations were within the range of 95.86% to 106.28%, with RSD < 13.2%, which revealed no obvious ion suppression or enhancement for the analytes in beagle dog plasma. The extraction recovery and matrix effect of IS were 96.81% and 98.74%, respectively. The data obtained are presented in Table 4.

#### 2.3.5. Stability 

The test data for the stability of the analytes at three concentrations under various conditions are presented in Table 5. The results indicate that all samples were stable under the conditions mentioned above, and that the stability of samples had not been affected during the analysis process.

### 2.4. Application to a Pharmacokinetic Study

**Figure 3:**
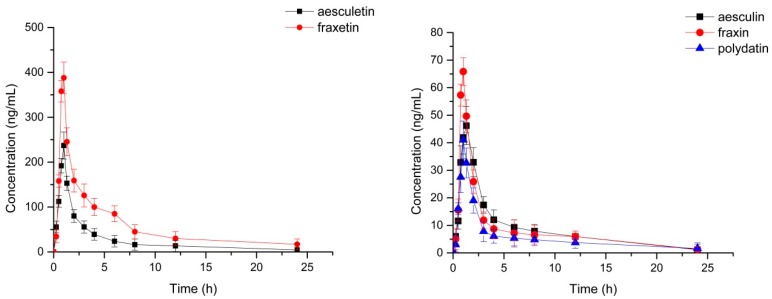
Mean plasma concentration–time profiles of aesculin; aesculetin; fraxetin; fraxin; polydatin in beagle dog plasma after oral administration of the extract of *L. palustre* (n = 6, Mean ± SD).

The validated UPLC-ESI-MS/MS method was successfully applied for the simultaneous determination of aesculin, aesculetin, fraxetin, fraxin and polydatin in beagle dog plasma after oral administration of the extract of *L. palustre* at a dosage of 0.27 g/kg. The pharmacokinetic parameters analyzed are presented in Table 6, and the mean plasma concentration–time profiles are illustrated in Figure 3. The LLOQ of aesculin was 0.50 ng/mL, 1.09 ng/mL for aesculetin, 2.15 ng/mL for fraxetin, 1.25 ng/mL for fraxin and 0.50 ng/mL for polydatin. The concentration of Cmax-to-LLOQ ratio of the analytes was greater than 20. The results indicated that the UPLC-MS/MS method has adequate sensitivity to measure the five components in beagle dog plasma.

The time to reach the maximum plasma concentration (T_max_) was 1.32 ± 0.38 h for aesculin, 1.03 ± 0.27 h for aesculetin, 0.94 ± 0.23 h for fraxetin, 0.83 ± 0.18 h for fraxin and 1.15 ± 0.15 h for polydatin. These results indicate that the absorption of aesculin might be slow in beagle dog plasma. The elimination half-life (T_1/2_) was 3.43 ± 0.47 h for aesculin, 4.25 ± 0.18 h for aesculetin, 3.76 ± 0.35 h for fraxetin, 2.99 ± 0.29 h for fraxin and 3.36 ± 0.31 h for polydatin. These results indicate that the elimination of fraxetin may be slow because of the longer T_1/2_ than the other four components.

The content of the five analytes was determined by HPLC in this paper. We can conclude that the content of aesculin was higher than aesculetin, and that fraxin was higher than fraxetin in the extract of *L. palustre*. However, the value of the last measurable plasma concentrations (AUC_0→t_) of aesculetin and fraxetin exceed those of aesculin and fraxin in Table 6. The maximum plasma concentrations (C_max_) of aesculin, aesculetin, fraxetin, fraxin and polydatin were 46.75 ± 7.46, 209.9 ± 27.65, 369.7 ± 48.87, 67.04 ± 12.09 and 47.14 ± 12.04 ng/mL, respectively; it can be seen that the C_max_ of fraxetin was higher than others. The phenomena above may be due to the fact that aesculin and fraxin can be transformed into aesculetin and fraxetin, respectively, in beagle dog plasma. According to the chemical structures of the analytes in Figure 1, aesculin and fraxin are the glucosides of aesculetin and fraxetin. Previous studies have revealed that aesculin can be completely metabolized to aesculetin under the action of gut bacteria, because it degrades the glycoside of aesculin to facilitate its absorption in the intestine [23]. Similar experimental results have also appeared in other reports about the metabolic fate of fraxinin, indicating that fraxin can be extensively metabolized to fraxetin in vivo, and that intestinal microflora play an important role in this process [24]. 

## 3. Materials and Methods

### 3.1. Chemicals and Reagents

The aesculin (151030) and fraxetin (151016) were purchased from Chengdu Pufei De Biotech Co., Ltd. (Chengdu, China). The aesculetin (15071508), fraxin (17041620), polydatin (170062102) and puerarin (17111007) (internal standard, IS) were purchased from Chengdu Mansite Biotech Co., Ltd. (Chengdu, China). The purity of reference standard was confirmed to be more than 98% by HPLC (Waters, Milford, MA, USA). The chemical structures of the analytes and IS were shown in Figure 1. Chromatographic-grade methanol and acetonitrile were obtained from Fisher Scientific (Waltham, NJ, USA). Deionized water was purchased from A.S. Watson Group Ltd. (Hong Kong, China). All other chemical reagents were analytical grade.

The medicinal herb *L. palustre* was collected from Daxinganling, Heilongjiang Province, China, and identified by Professor Zhenyue Wang of Heilongjiang University of Chinese Medicine.

### 3.2. Instruments and Chromatographic Conditions 

A Waters Acquity UPLC system (Waters, Milford, MA, USA) was used for liquid chromatography analysis. Chromatographic separation was performed on an Acquity UPLC HSS T3 C18 column (2.1 mm × 100 mm, 1.8 μm) with a mobile phase composed of 0.1% formic acid (A) and acetonitrile (B). The gradient elution mode at a flow rate of 0.4 mL/min was as follows: 0 min at 2% B, 0–3 min at 2%–80%, 3–5 min at 80%–2%. The column temperature was maintained at 25 °C. Samples were analyzed by injecting 5 μL into the instrument, with a total run time of 5 min.

An AB Sciex 4000 QTRAP MS (AB Sciex, Foster, CA, USA) equipped with an electrospray ionization interface (ESI) was used for mass spectrometry detection. The quantitative analysis of analytes was performed in the negative mode by multiple reaction monitoring (MRM, AB Sciex, Foster, CA, USA), with ion transitions of *m*/*z* 339.1–*m*/*z* 176.8 for aesculin, *m*/*z* 176.8–*m*/*z* 88.9 for aesculetin, *m*/*z* 206.8–*m*/*z* 192.1 for fraxetin, *m*/*z* 369.1–*m*/*z* 206.9 for fraxin, *m*/*z* 389.1–*m*/*z* 227.0 for polydatin and *m*/*z* 415.2–*m*/*z* 295.1 for puerarin. The mass scan spectra of the analytes and IS were shown in Figure 1. Mass spectrometric parameters were optimized for better ionization of analytes. The optimal MS parameters were as follows: source temperature at 450 °C, capillary voltage at 3200 V, Nitrogen was used as desolvation and cone gas with a flow rate of 700 L/h and 150 L/h, cone voltage at 30 V.

### 3.3. Preparation of L. palustre Extract

The *L. palustre* was extracted by refluxing with ethanol three times, each time for 1 h, then filtrated with gauze. The filtrate was combined and concentrated up to dryness. Based on measurement, we could see that the extraction rate was 23%. According to the conversion relationships between humans and animals, the oral dosage for dogs was determined to be 0.27 g/kg. The contents of aesculin, aesculetin, fraxetin, fraxin and polydatin in *L. palustre* were determined by HPLC (Waters). The content of aesculin was 5.9 mg/g, 0.9 mg/g for aesculetin, 2.1 mg/g for fraxetin, 2.3 mg/g for fraxin and 7.4 mg/g for polydatin in extracts of *L. palustre*, respectively. The extracts were fed to beagle dogs in the form of capsules. 

### 3.4. Preparation of Calibration Standards and Quality Control (QC) Samples 

A mixed stock solution was prepared in methanol and the concentration of the analytes was 97.50 ng/mL for aesculin, 209.2 ng/mL for aesculetin, 412.8 ng/mL for fraxetin, 241.0 ng/mL for fraxin, and 97.50 ng/mL for polydatin, respectively. A 260.0 ng/mL working solution of IS was prepared by diluting a stock solution with methanol. The mixed calibration standard solutions for aesculin (0.50, 1.52, 3.04, 6.09, 12.18, 24.37, 48.75, 97.50 ng/mL), aesculetin (1.09, 3.27, 6.54, 13.08, 26.16, 52.32, 104.6, 209.2 ng/mL), fraxetin (2.15, 6.45, 12.90, 25.80, 51.60, 103.2, 206.4, 412.8 ng/mL), fraxin (1.25, 3.76, 7.53, 15.06, 30.12, 60.25, 120.5, 241.0 ng/mL) and polydatin (0.50, 1.52, 3.04, 6.09, 12.18, 24.37, 48.75, 97.50 ng/mL) were prepared by further dilution of the mixed stock solution with methanol. Quality control (QC) samples at low, middle and high concentrations were 1.52, 12.18, 78.00 ng/mL for aesculin, 3.27, 26.16, 167.3 ng/mL for aesculetin, 6.45, 51.60, 330.2 ng/mL for fraxetin, 3.76, 30.12, 192.8 ng/mL for fraxin, and 1.52, 12.18, 78.00 ng/mL for polydatin. All working solutions were stored at 4 °C until use.

### 3.5. Sample Preparation 

Protein precipitate method was used for treatment of the biological plasma samples. 100 μL of IS and 300 μL of methanol were added to a 100 μL plasma sample in a 10 mL glass tube. The tube was vortexed (Qingpu Huxi instrument Factory, Shanghai, China) for 3 min and centrifuged (Anhui USTC Zonkia Scientific Tnstruments Co., Ltd., Anhui, China) at 3500 rpm for 10 min. Supernatant was transferred to a new glass tube and evaporated to dryness under nitrogen stream at 40 °C. The residue was reconstituted with 100 μL of initial mobile phase, then vortexed for 3 min and centrifuged at 12,000 rpm for 10 min. A 5 μL aliquot was injected into the UPLC-ESI-MS/MS system for analysis.

### 3.6. Method Validation 

Method validation was implemented in beagle dog plasma according to the US Food and Drug Administration Bio-analytical Method Validation Guide [25]. The test projects include selectivity, linearity, sensitivity, precision, accuracy, matrix effect, recovery, stability.

#### 3.6.1. Selectivity and Specificity 

The specificity of the method was estimated by comparing the chromatograms of the blank plasma from six beagle dogs, spiked plasma samples with the analytes and IS at LLOQ level, spiked plasma samples with the analytes and IS at QCM level, and plasma samples from beagle dog 1 h after oral administration of the extract of *L. palustre*. The purpose of specificity research was to detect whether endogenous interference from other substances has an effect on retention time of analytes and IS.

#### 3.6.2. Linearity and Sensitivity 

The linearity of the five compounds was evaluated via construct regression equation. Calibration curve was established by plotting peak area ratios of the analytes to IS versus plasma at eight concentration levels in the linear range of analytes, using the least-square linear regression with weighting factor 1/X^2^. The regression equation was Y = aX + b.

The lowest limit of quantification (LLOQ) was defined as the lowest concentration in the calibration curve at which the signal-to-noise (S/N) ratio was about 10. The precision and accuracy of LLOQ should be within acceptable limits. The precision was required to be less than 20% and the accuracy was required to be less than ±20%.

#### 3.6.3. Precision and Accuracy 

The precision and accuracy were evaluated by analyzing six replicates of QC samples at three concentration levels. The intra-day and inter-day precisions were determined on the same day and on three consecutive days. Precision and accuracy were represented by RSD (%) and RE (%), respectively. Furthermore, the values of precision were required not to exceed 15%, and the accuracy should be better than ±15%. 

#### 3.6.4. Extraction Recovery and Matrix Effect 

The extraction recovery and matrix effect were determined in six replicates at three QC concentrations. Extraction recovery of analytes was evaluated by comparing the peak areas of the analytes from the extracted QC samples with those samples spiked after extraction of blank plasma at equivalent concentrations. The matrix effect of the analytes was assessed by comparing the peak areas of the analytes obtained from the plasma samples with those extracted from standard solutions at the same concentration. The extraction recovery and matrix effect of IS was also determined in a similar way at a single concentration of 260.0 ng/mL.

#### 3.6.5. Stability

The stability of the analytes in beagle dog plasma was measured by analyzing six replicates of QC samples at three concentration levels in various storage conditions, including short-term stability (kept at room temperature for 24 h), long-term stability (kept at −20 °C for 2 weeks), three freeze/thaw cycles stability (repeated freeze/thaw for three cycles from −20 °C to room temperature) and post-preparation stability (processed samples kept under the auto-sample conditions for 24 h). Samples were considered stable if the deviation was within 15% compared with a freshly prepared solution.

### 3.7. Application to a Pharmacokinetic Study

#### 3.7.1. Animals 

Six healthy beagle dogs (6 female; weight, 12–14 kg) were provided by Shenyang Kangping Institute of Laboratory Animal (SCXK (Liao) 2014-0003). The dogs were kept with adequate food and water under constant temperature and humidity, except for fasting 12 h before the experiment with free water. The 0.25 mL plasma samples were collected in heparinized tubes pre-dose and at 0.25, 0.50, 0.75, 1.0, 1.3, 1.67, 2.0, 3.0, 4.0, 8.0, 12.0 and 24.0 h after oral administration of the extracts of *L. palustre* at a dosage of 0.27 g/kg. The plasma was centrifugated at 3500 rpm for 10 min at 4 °C, and the supernatant was collected into a 1.5 mL tube before being stored at −20 °C.

#### 3.7.2. Data Analysis 

DAS 2.0 (Shanghai, China) with a non-compartmental model was used to acquire pharmacokinetic parameters. The values of maximum plasma concentration (C_max_) and time to reach the maximum plasma concentration (T_max_) were observed from the mean plasma concentration–time profiles. The terminal elimination rate constant (K_e_) was measured by linear regression of the terminal log-linear part of the mean plasma concentration-time curve, and the T_1/2_ = 0.693/K_e_ was used to measure elimination half-life (T_1/2_). The area under the plasma concentration-time curve at the time from 0 to the last measurable plasma concentration (AUC_0→t_) was estimated by using the linear trapezoidal rule. The formula of AUC_0→∞_ = AUC_0→t_ + C_t_/K_e_ can be used for calculating the area under the plasma concentration-time curve at any time from 0 to infinity (AUC_0→∞_). All analysis results were expressed as mean ± SD. 

## 4. Conclusions 

In this paper, a rapid, sensitive and selective UPLC-ESI-MS/MS method was successfully applied for simultaneous determination of aesculin, aesculetin, fraxetin, fraxin and polydatin in beagle dog plasma. This method provided adequate recovery and matrix effect with precision, accuracy and lower detection limits. All test results are within acceptable limits with a total run time of 4.5 min. This is the first report about the pharmacokinetics of aesculin, aesculetin, fraxetin, fraxin and polydatin in beagle dog plasma. Therefore, the information obtained provides a reference for further clinical application of *L. palustre*.

## Figures and Tables

**Table 1:** Optimized mass spectrometric parameters for analytes and IS.

Analytes	Precursor Ion (*m*/*z*)	Product Ion (*m*/*z*)	Declustering Potential (V)	Collision Energy (V)	Polarity
aesculin	339.1	176.8	103.1	40.7	negative
aesculetin	176.8	88.9	69.3	34.9	negative
fraxetin	206.8	192.1	64.9	22.6	negative
fraxin	369.1	206.9	75.8	30.8	negative
polydatin	389.1	227.0	85.6	22.0	negative
puerarin	415.2	295.1	92.9	34.4	negative

**Table 2:** Regression equations, correlation coefficients and linear ranges of analytes in beagle dog plasma.

Analytes	Regression Equations	R	Linear Range (ng/mL)	LLOQ (ng/mL)
aesculin	Y = 2.62 × 10^−3^X − 0.34 × 10^−2^	0.9988	0.50–97.50	0.50
aesculetin	Y = 0.71 × 10^−3^X + 2.24 × 10^−2^	0.9985	1.09–209.2	1.09
fraxetin	Y = 1.80 × 10^−3^X − 0.65 × 10^−2^	0.9983	2.15–412.8	2.15
fraxin	Y = 1.53 × 10^−3^X − 0.16 × 10^−2^	0.9986	1.25–241.0	1.25
polydatin	Y = 7.60 × 10^−3^X + 1.12 × 10^−2^	0.9981	0.50–97.50	0.50

**Table 3:** Precision and accuracy of analytes at LLOQ and QC samples in beagle dog plasma (n = 6).

Analytes	Spiked Concentration (ng/mL)	Mean ± SD (ng/mL)	Intra-day Precision RSD (%)	Inter-day Precision RSD (%)	Accuracy RE (%)
aesculin	0.50	0.56 ± 0.15	10.7	6.9	16.5
	1.52	1.54 ± 0.22	4.1	8.4	1.6
	12.18	12.42 ± 2.16	9.5	6.2	2.8
	78.00	74.11 ± 6.30	5.9	4.6	6.8
aesculetin	1.09	1.12 ± 0.15	11.9	14.2	5.1
	3.27	14.02 ± 1.57	6.0	7.4	2.4
	26.16	24.31 ± 2.23	8.9	9.1	−4.9
	167.3	172.3 ± 18.35	3.5	8.9	5.4
fraxetin	2.15	2.08 ± 0.22	15.2	7.7	−1.8
	6.45	6.34 ± 0.36	4.5	3.3	−1.5
	51.60	53.53 ± 2.31	6.4	7.2	3.7
	330.2	347.9 ± 18.46	9.3	7.9	5.4
fraxin	1.25	1.20 ± 0.11	10.5	16.1	−3.4
	3.76	3.44 ± 0.36	5.8	3.9	−5.2
	30.12	32.90 ± 2.74	7.0	5.6	5.9
	192.8	186.3 ± 11.06	5.8	7.3	3.3
polydatin	0.50	1.23 ± 0.13	6.2	15.6	13.1
	1.52	1.55 ± 0.39	9.5	11.6	8.3
	12.18	13.87 ± 2.67	6.9	5.8	6.4
	78.00	75.1 ± 13.39	4.3	7.7	4.8

**Table 4:** Extraction recovery and matrix effect of analytes in beagle dog plasma (n = 6).

Analytes	Spiked Concentration (ng/mL)	Extraction Recovery		Matrix Effect	
		Mean (%)	RSD (%)	Mean (%)	RSD (%)
aesculin	1.52	85.25	9.3	97.08	2.4
	12.18	86.11	6.9	106.28	4.8
	78.00	91.59	5.2	100.59	6.4
aesculetin	3.27	89.14	10.2	97.13	13.2
	26.16	91.15	5.2	96.87	7.6
	167.3	87.10	6.9	100.91	7.0
fraxetin	6.45	86.65	8.9	99.51	10.8
	51.60	98.54	6.8	101.08	7.4
	330.2	88.21	5.2	106.22	8.1
fraxin	3.76	80.10	5.9	98.66	8.3
	30.12	88.58	6.1	100.39	5.5
	192.8	90.20	3.4	102.52	6.8
polydatin	1.52	82.07	7.7	95.86	6.4
	12.18	81.33	5.2	99.28	7.6
	78.00	88.24	3.3	100.23	5.2
IS	260.0	96.81	5.9	98.47	8.9

**Table 5:** Stability of analytes in beagle dog plasma under various conditions (n = 6).

Analytes	Spiked Concentration (ng/mL)	Stability (RE %)			
		Long-Term	Short-Term	Three Freeze-Thaw	Post-Preparation
aesculin	1.52	4.3	−5.5	2.7	3.8
	12.18	5.6	4.1	2.4	−4.1
	78.00	−2.7	−7.5	−2.5	3.3
aesculetin	3.27	−1.9	2.0	4.9	6.7
	26.16	5.3	−4.9	−3.4	2.5
	167.3	5.8	3.1	5.3	4.7
fraxetin	6.45	3.7	4.3	8.7	4.1
	51.60	7.7	5.1	7.2	10.7
	330.2	7.5	6.3	−4.5	5.3
fraxin	3.76	6.4	2.7	7.6	3.4
	30.12	5.9	6.4	−2.5	8.4
	192.8	4.6	7.8	−4.3	6.9
polydatin	1.52	2.9	3.3	7.6	2.4
	12.18	3.8	6.9	5.9	−2.7
	78.00	8.4	5.7	−4.3	3.7

**Table 6:** Pharmocokinetic parameters of five analytes in beagle dog plasma after oral administration of the extract of *Ledum palustre* L.

Analytes	C_max_ (ng/mL)	T_max_ (h)	T_1/2_ (h)	AUC_0→t_ (ng/h/L)	AUC_0→∞_ (ng/h/L)
aesculin	46.75 ± 7.46	1.32 ± 0.38	3.43 ± 0.47	258.5 ± 20.45	342.4 ± 35.82
aesculetin	209.9 ± 27.65	1.03 ± 0.27	4.25 ± 0.18	314.3 ± 30.92	355.9 ± 30.52
fraxetin	369.7 ± 48.87	0.94 ± 0.23	3.76 ± 0.35	940.1 ± 52.89	992.8 ± 46.96
fraxin	67.04 ± 12.09	0.89 ± 0.18	2.99 ± 0.29	147.2 ± 21.74	191.5 ± 22.18
polydatin	47.14 ± 12.04	1.15 ± 0.15	3.36 ± 0.31	89.82 ± 14.04	98.28 ± 12.11
